# Inflammaging and Immunosenescence as Part of Skin Aging—A Narrative Review

**DOI:** 10.3390/ijms24097784

**Published:** 2023-04-24

**Authors:** Justyna Pająk, Danuta Nowicka, Jacek C. Szepietowski

**Affiliations:** Department of Dermatology, Venereology and Allergology, Wrocław Medical University, 50-368 Wrocław, Poland

**Keywords:** skin aging, inflammaging, immunosenescence, immunity, immune system, risk factors

## Abstract

Inflammaging and immunosenescence are associated with aging of the human body, but there are key differences between them. Immunosenescence aims to adapt the body systems to aging, while inflammaging is considered a consequence of immunosenescence. There has been much research in the area of immunosenescence and inflammaging recently, yet our understanding of aging and the ability to develop interventions to decrease the harmful effect of aging on the human body is insufficient. This review is focused on immunosenescence and inflammaging processes in the skin. We aimed to identify factors that influence inflammaging, skin aging, and their mechanisms. We discussed the role of triggering factors (e.g., UV radiations, changes in bioavailability of nitric oxide, senescence-associated secretory phenotype factors, and reactive oxygen species) and inhibiting factors that can potentially be used as anti-aging treatments, as well as the idea of geroprotectors and senotherapeutics. We concluded that while knowledge on external factors can help people to improve their health conditions, knowledge on biochemical factors can help researchers to understand inflammaging process and develop interventions to minimize the impact of aging on the human body. Further research is needed to better understand the role of factors that can slow down or accelerate inflammaging.

## 1. Introduction

Invisible, slow, sterile and symptom-free processes of inflammation progressing with time take place in all organs of the human body [1]. Along with aging, the immune system adapts; some immune processes change, while others weaken or even lose the capacity to respond properly to external and internal factors. The phenomenon of chronic inflammation is called immunosenescence or even inflammaging [2,3]. Inflammaging that occurs in the skin can lead to tissue damage, cancer or other skin diseases associated with aging. Furthermore, chronic exposure to inflammatory stimulation, together with some specific characteristics of the human genome, increases susceptibility to age-related diseases such as atherosclerosis, Alzheimer’s disease, osteoporosis, and diabetes [1].

In the literature, two terms are used to describe immune changes with aging, namely, immunosenescence and inflammaging. The concept of immune system aging was introduced in the 1980s when researchers identified changes in innate immunity caused by aging that can cause many age-related diseases. Inflammaging and immunosenescence are considered by some researchers to be almost identical, but there are some key differences between them. Immunosenescence aims to adapt the body systems to aging, while inflammaging is considered a consequence of immunosenescence [4]. However, this inflammaging cannot be separated from immunosenescence—one drives the other and vice versa; however, they are equally responsible for skin aging [5]. Over the last decade, there has been much research in the area of immunosenescence and inflammaging, yet our understanding of aging and the ability to develop interventions to decrease the harmful effect of aging on the human body is insufficient. This review is focused on immunosenescence and inflammaging processes in the skin. We aimed to identify factors that influence inflammaging, skin aging, and their mechanisms. As aging is an unavoidable process, having that knowledge can help us to react in time, slow down aging, or even partially prevent it.

## 2. Overlap of Inflammaging and Immunosenescence

**Figure 1:**
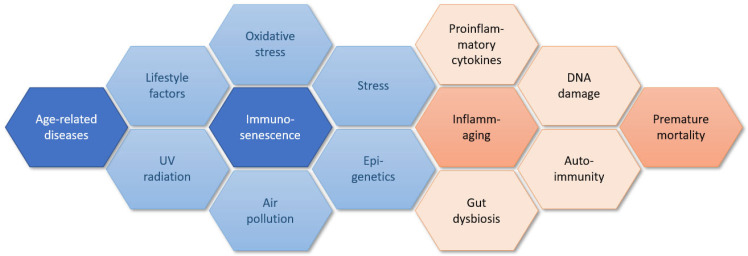
Factors contributing to immunosenescence and inflammaging.

Inflammaging is defined as sterile, slowly progressing, low-grade inflammation which is a normal process of aging and cannot be separated from aging of the immune system which is called ‘immunosenescence’ [6,7,8,9,10,11,12,13,14,15,16,17,18,19]. However, to date, it is unclear which of these is the reason and which is the cause [10]. In Figure 1, we present a complex interplay of a body of factors that contributing to immunosenescence and inflammaging that are discussed further in the paper.

Inflammaging is not only an intrinsic process caused by aging that results in signs such as telomere shortening, genomic instability and mitochondrial dysfunction, or stem cell exhaustion [12,18]. It is also combined with some external environmental factors, in particular pathogens, ultraviolet (UV) radiation, smoking, and air pollution [8,9,10]. One of the theories claims that when the human body loses its natural ability to cope with various extinct stressors, autoreactivity increases what is linked with immunosenescence and leads to accelerated aging, development of various diseases, tissue damage, and increased mortality. Some processes that underlie inflammaging have been identified. They include cellular aging, inflammasome activation, mitochondrial dysfunction, autophagy, and mitophagy, ubiquitin-proteasome system, and DNA damage. Furthermore, several signaling pathways are involved in the regulation of inflammaging. Most often described are the NF-κB signaling pathway, mTOR signaling pathway, RIG-I signaling pathway, NOTCH signaling pathway, TGF-β signaling pathway, RAS signaling pathway, and regulation of sirtuin activity [18].

Various authors mention the key role of an increased level of pro-inflammatory cytokines, also noting the importance of maintaining a correct balance between them. Over time, cells such as macrophages, neutrophils, and natural killer (NK) cells, namely, the innate immune system, become dominant whereas the adaptive immune system, namely, B and T cells responsible for immunosenescence gradually play a smaller role [15]. Immune response processes are beneficial at a young age, but later can bring more harm to human cells. It is important to know the difference between temporary acute inflammation and very slow low-grade inflammation. The first (when its level is accurate) is responsible for defending from infections while the second is responsible for inflammaging [14].

A great number of authors observe the key role of microbiota and oxidative stress in inflammaging [10,11,12,13,14,15]. Furthermore, a senescence-associated secretory phenotype (SASP) is believed to be responsible for having a negative influence on senescent cells. Therefore, the occurrence of SASP contributes to increasing pro-inflammatory cytokines levels and finally drives inflammaging. This is probably associated with declining immunosurveillance with time and is caused by the permanent activation of the DNA response to damaging paths [10,11,18]. Several studies have showed a direct link between skin aging and the development of age-related chronic diseases such as Alzheimer’s disease, atherosclerosis, diabetes, cancers, and age-related macular degeneration and skin aging [11,12,13,15,18,20]. Moreover, there are papers indicating that inflammaging-related cytokines provoke type 2 inflammatory responses and predispose to pruritic dermatoses; however, the authors also point out that evidence from existing studies is insufficient and this field needs further investigation [14]. Skin inflammaging in particular is of great importance because the skin is the largest organ and the first barrier against external hazards; therefore, it needs to be safeguarded as much as possible to maintain its protective function. Furthermore, the skin microbiome is also an important factor that can influence inflammation. Studies on mice showed that improving skin function leads to lower cytokine levels in the whole organism [15,21].

## 3. Process of Inflammaging

In the literature, several processes taking place in skin inflammaging have been described (briefly summarized in Table 1):

- Excessive secretion of TNF-α, IL-6, IL-1β, and IL-2 [9,10,14,15,18,20,22,23,24].
- Excessive secretion of IL-1 and IL-18 [18,23,24,25,26].
- Elevated level of E2 prostaglandin [24].
- Excessive mitochondrial division leading to overproduction of reactive oxygen species (ROS) and inflammatory factors [22].
- Accumulation of senescent cells [22].
- Elevated level of C-reactive protein [9,14,15,18,23].
- Elevated level of metalloproteinases (MMP1, MMP3, MMP10, MMP12) [27].

**Table 1:** Factors influencing inflammaging.

Factors Accelerating Inflammaging	Factors Slowing down Inflammaging	Factors with Variable Influence on Inflammaging
oligomeric α-Synuclein [28] UV radiation [9,10,11,12,13,19,27,29] PM [10,11,21,23] ROS/oxidative stress [10,11,12,13,15,21,27] tobacco extract [11,21] excessive nutrient supply [12] living in urban areas [21]	three ingredient blends (probably only in vitro, need more long-term studies) [25] bakuchiol and vanilla tahitensis extract [30] curcumin [8,21] aloe vera topically [21] resveratrol [21] AdipoRon [22] topical nanoemulsion formulation containing tocotrienol-rich fraction [9] *Centella asiatica* extract [26] estrogen [24] rapamycin topically (potentially) [10] (in [14] no beneficial effects) metformin topically [10,14] emollients/moisturizers (preventing epidermal disruption) [16] seaweed extract (*Laminaria Japonica*) [17]	microbiota (i.e., skin, gut) [6,13,14,15] epidermal function (dysfunction pro-inflammaging, normal function anti-inflammaging) [31] diet [10]: excess of nutrients—pro-inflammaging, starvation—anti-inflammaging [12], anti-inflammaging when appropriate [32]

AdipoRon, adiponectin receptor agonist; PM, particulate matter; ROS, reactive oxygen species; UV, ultraviolet.

## 4. Triggering Factors

Elements that probably accelerate inflammaging are UV radiation from photo exposure [9,29]; oligomeric α-Synuclein (in connection with neurodegenerative diseases or through accumulation with age or caused by the sun) leading to inflammation and decreased keratinocytes proliferation and regeneration [28]; oxidative stress that prematurely causes aging of senescent cells by damaging their DNA [7] and stimulating metalloproteinases to damage elastin and collagen in the skin [21]. UV radiation, especially UV B, causes both inflammation and immunosuppression (locally in the skin and also systemic) but in a different mechanism than during chronological aging [33]. Moreover, UV radiation drives fibroblasts to produce more pro-inflammatory cytokines such as IL-1 and TNF-α and mast cells to produce inflammatory mediators such as prostaglandins and leukotrienes [13]. These are called senescence-associated secretory phenotype (SASP) factors. SASP factors are excreted particularly intensively in the environment of senescent fibroblasts, which is important in the skin as they accelerate skin aging due to chronic inflammation and degradation of extracellular matrix as visible in photoaging as well [14,22]. Furthermore, UV radiation can also boost inflammaging through elevating metalloproteinase levels [27]. Solar UV irradiation significantly elevates the level of the matricellular cysteine-rich protein 61 (CCN1) in photo-exposed human skin. CCN1 then triggers the rise of IL-1β, which is the most common pro-inflammaging cytokine. It can also inhibit collagen production on its own by increasing metalloproteinase levels [34]. Another study showed that M1 to M2 macrophage imbalance in photo-exposed human dermis move to M1, although the total number of macrophages did not change. This also correlates with a decreased level of IL-34 in aged skin and leads to inflammaging [35].

Vascular aging involves all human organs, including the skin. Aging-associated endothelial dysfunction is a complex process with several major alterations being involved. With time, bioavailability of nitric oxide (NO) becomes reduced caused by lower vascular expression and activity of nitric oxide synthase (NOS) enzymes. Altered balance between vascular relaxation and nitrosative stress as NO is considered to play a role in longevity and cardiovascular health when it is lower [36]. NO-dependent mechanisms of skin aging have been confirmed to contribute to subcutaneous vasodilatation in aged women which in turn contribute to changes in the oxidative processes in the skin and accelerated aging [37].

Numerous studies have shown that thyroid hormones play a role in the regulation of dermal fibroblasts during aging [38]. A study by Gunin and Golubtsova showed that the total number of fibroblasts in the dermis and their reduced proliferation is related to a decrease in the level of thyroid hormone receptors α and β in dermal fibroblasts. The number of thyroid hormone receptors is the highest after birth and then decreases until 40 years of age and becomes stable after this. The authors concluded that changes in the number of thyroid hormone receptors are correlated with age [39]. Although thyroid dysfunction coexists with many skin conditions, the role of thyroid hormone receptors in skin diseases and their potential use as therapeutical targets require further research [38]. Tobacco extract is another accelerating factor that works by increasing oxidative stress, which can also trigger the secretion of pro-inflammatory cytokines and metalloproteinases [11]. Living in an urban environment and smoking are independent factors leading to premature skin aging [21]. Particulate matter in the air is a factor that has a direct (by AhR activation, ROS generation, phosphorylation of p38 MAPKs in epidermal cells, and DNA damage) and indirect accelerating influence on inflammaging in the dermis [21,23]. It can also cause various skin disorders (atopic dermatitis, urticaria, contact dermatitis, psoriasis, skin cancer, acne) if exposure is chronic or occurs at a young age [21]. Finally, even reduced ambient humidity and excessive psychological stress can induce and enhance inflammaging [20].

## 5. Geroprotectors and Senotherapeutics

Vitamins and other micronutrients can act as a shield against aging as they have the ability to affect cell function [40] Currently, the term senotherapy has been introduced as part of anti-aging therapy. It develops approaches that can eliminate or prevent cell senescence. As such, senotherapy is focused on improving healthy aging, which translates into postponing natural aging and delaying the onset of age-related diseases. Senotherapeutics are divided into two categories: senolytics and senomorphics. Senolytics aim to eliminate senescent cells through apoptosis and, in principle, can be administered as treatments at certain time intervals. Conversely, senomorphics can help diminish the effects of senescent cells by suppressing their ability to secrete factors that affect neighboring cells. Senomorphics should be administered continuously to maximize their effect [41]. To date, much evidence has been gathered from experimental studies. However, although such types of therapeutics are interesting and there are many ongoing studies in this field, none of the medicinal products indicated for slowing down aging have been approved [40]. Both senolytics and senomorphics are factors that inhibit senescence and inflammaging.

The definition of gerontoprotectors is less clear; however, the term has been used in the literature. Most often, this group of products is defined as substances that try to counteract low-grade inflammation that develops during aging [42]. Currently, there are several drugs on the market that are believed to act as gerontoprotectors, including metformin, rapamycin, or ruxolitinib. Metformin is a first-line drug for treating type 2 diabetes with beneficial effects on glucose metabolism. In addition to this action, it has been shown to reduce microvascular risk and the emergent risk of myocardial infarction and death from any cause [43]. A meta-analysis that focused on excluding confounders such as body mass index, study type, and time-related biases, revealed that metformin may reduce cancer incidence and mortality in patients with diabetes [44]. One of the mechanisms behind additional properties of metformin is its ability to block the expression of genes coding for multiple inflammatory cytokines seen during cellular senescence, that is, the NF-κB pathway [45]. The recent Metformin in Longevity Study (MILES) trial, a randomized cross-over study, was conducted to investigate whether the use of metformin can result in changes in the transcriptome. However, it has not been elucidated if this additional effect of metformin is due to its anti-hyperglycemic and insulin-sensitizing properties which can secondarily reduce the risk of lifestyle diseases and further improve health span [46]. Rapamycin (sirolimus), which inhibits the activation of the mTOR signaling pathway, acts as an immunosuppressant and is used to suppress the immune system after kidney transplantation. In addition to this action, rapamycin has been shown to prevent lymphoma and other types of cancer, including skin cancer, in patients receiving transplants [47]. In numerous studies, rapamycin has showed an effect on immune system functioning (e.g., concentrations of plasma immunoglobulin, frequency of specific T cell subsets, concentrations of cytokines in blood and heart, response to vaccination); positive changes in body traits related to aging (body weight, fat mass, lean mass, thyroid follicle size, cardiac dimension, heart weight); and neurobehavioral changes (motor activity, learning, and memory) [48]. However, it has not been yet elucidated whether those effects are symptomatic or whether the drug can truly slow down the aging process. Another drug, ruxolitinib, a selective JAK1/2 inhibitor, is used in oncology to treat patients with myelofibrosis and polycythemia as well as to treat patients with graft-versus-host disease. Biochemical research shows that the JAK pathway is activated in adipose tissue over time. Furthermore, by initiating the JAK pathway, SASP secretion can be diminished. This rationale is behind the use of JAK1/2 inhibitors which have been shown to reduce inflammation and frailty in animal models [49]. Benefits in terms of reduced systemic inflammation, increased physical capacity, metabolic functions, muscle regeneration, and regained adipose tissue homeostasis achieved with JAK1/2 inhibitors show their effect on alleviating various age-related symptoms [50]. Although the benefits of using those agents are promising, it has to be remembered that their use as anti-aging medication requires further research. Furthermore, the strong systemic effect of these drugs will limit their use related to skin inflammaging.

Preparations of vitamin D (colecalciferol or cholecalciferol) are indicated for the treatment and prevention of vitamin D deficiency; however, this vitamin is a fat-soluble steroid molecule. Its active form is mainly synthesized in the kidneys following skin exposure to UV B radiation. Vitamin D3 exerts many effects in the human body [51]. Vitamin D insufficiency manifests itself in bone defects and an increased risk of developing many lifestyle diseases, including neurological diseases, obesity, diabetes and metabolic disorders, cardiovascular diseases, autoimmune diseases, infections, or even cancers [52]. Low levels of vitamin D are associated with poor self-reported health status, especially in the elderly [53]. The multipotential effect of vitamin D is rooted in its ability to downregulate or suppress the biochemical pathways that are involved in the processes of inflammation and cellular aging. Vitamin D has a strong impact on the functioning of the immune system. Most cells involved in the immune system responses express a specific vitamin D receptor and respond to vitamin D with an impact on biochemical pathways [54]. For example, by a physical interaction between the vitamin D receptor and IκB kinase β (IKKβ), which can be upregulated by vitamin D, the expression of NF-κB becomes downregulated along with the blockage of p65 translocation. This action leads to a reduction in inflammation and aging processes [52]. An increased level of activation of NF-κB is observed in all organs, including skin. Animal models show that the disruption of the vitamin D receptor alters interfollicular epidermal differentiation and hair follicle cycling, and increases the risk of skin cancer formation [55].

## 6. Factors That Inhibit Inflammaging

The constant interest in staying young as long as possible drives research on finding new products to stop inflammaging. Elements that probably slow down that process are bakuchiol and *Vanilla tahitensis* extract based on studies performed in vitro and ex vivo skin models. They reduce the expression of IL-8 and p-16, which could play an important role in communication with dermal endothelial cells, increasing the migration of neutrophils into the dermis and boosting inflammation) [30]. There are also studies in which the authors linked p-16 deficiency with family longevity and a lower number of age-associated diseases, such as dementia, arthritis, and type 2 diabetes [10]. Gruber et al. suggested that the three ingredients blend and can affect in vitro inflammaging by modulating the expression of active Caspase-1 in inflammasome-activated NHEK, which are key to the process of inflammaging by triggering the release of reservoirs of IL-1 and IL-18, leading later to a release of T cells and dendritic immune response cells. The authors pointed out that these results need to be confirmed by long-term studies [25]. Vollono et al.’s article reviewing several studies, showed that a potential anti-inflammatory and anti-oxidant effect could be achieved by topical use of curcumin, which may be due to its ability to reduce levels of TNF-α by activated macrophages, inhibit phosphorylase kinase, reduce the number of IL-17A, IL-17F, IL-22 and other pro-inflammatory cytokines, a high level of which has been observed in diseases with underlying inflammaging processes [8].

In a study conducted by Sun et al., it was found that the epidermis is the place in the skin where increased inflammatory factors (products of inflammaging) are mainly distributed. Moreover, they pointed out that AdipoRon (adiponectin receptor agonist) inhibited mitochondrial oxidative stress and inflammation by suppressing the excessive mitochondrial division that happens during skin inflammaging [22]. Another factor that can have anti-inflammaging properties is probably topical nanoemulsion formulation containing the tocotrienol-rich fraction by reducing increased by inflammaging levels of IL-6, IL-8 [9]. Maramaldi et al. presented results of their studies in which they showed that purified *Centella asiatica* extract reduces expression of IL-1β (a pro-inflammaging cytokine) and also protects DNA by decreasing thymine photodimerization [26]. There is also a study by Kovacts et al. in which they showed that this estrogen can potentially be used at low levels to reduce inflammaging by lowering the level of IL-6 [24]. Rapamycin applied topically reduces p16 expression and can partially inhibit fibroblast senescent and reduce oxidative stress [10]. Some studies report that aloe vera and resveratrol may have a beneficial influence on inflammaging by inhibiting NLRP3 inflammasome which stimulates the production of pro-inflammatory IL-1β cytokine [21]. Seaweed extract (*Laminaria japonica*) is another factor that has been shown to inhibit inflammaging in the skin. Its mechanism of action involves, in particular, blocking the inflammasome and NF-κB pathways and decreasing ROS and pro-inflammatory cytokine level [17].

There are factors that show variable influence on inflammaging. These are microbiota [6], epidermal function [31], and diet [12]. Identified evidence is presented in Table 1. The microbiota of the skin or gut can change in dysbiota with age turning from an efficient passenger (or so called metaorganism) to a pathogen that induces progress of inflammaging or even a disease [6,56]. However, not only does aging change our microbiota. There are many factors such as food, vaccines, antibiotics, living in an urban environment or particular human behaviors such as hygiene, more Cesarean sections, or replacing breastfeeding with modified milk [6,56]. Studies also showed the changes in microbiota with aging—predominating phyla of the skin—from *Actinobacteria* (i.e., *Propionibacterium*) to *Firmicutes* (i.e., *Staphylococcus*) and that a lower level of *Lactobacillus* on vulvovaginal mucosa can lead to vulvovaginal atrophy [56].

## 7. Inflammaging and Immunosenescence as Part of Skin Aging

A study conducted by Wen et al. presented several conclusions—(1) inflammaging depends on changes in epidermal function, (2) epidermal dysfunction elevates pro-inflammatory cytokines in both the skin and serum, and (3) restoring epidermal functions (i.e., by topical emollients) reduces pro-inflammatory cytokine levels (TNF-α, IL-1β, IL-6) [20,31]. Emollients are believed to maintain normal epidermal function and decrease pro-inflammatory cytokine levels [20]. Excessive nutrient delivery has an increasing influence on skin inflammaging due to increased accumulation of M1-type macrophages which have pro-inflammatory properties and a decreased amount of anti-inflammatory M2-types that are key cells in normal wound healing [12,35]. On the other hand, an appropriate diet that includes a poly-phenol-rich substances from fruits, vegetables, herbs, ω-3 fatty acids and vitamins is believed to have anti-inflammatory effects [32].

Some processes that take place during skin aging should be mentioned. They include degeneration and loss of collagen elastin fibers, both in the dermis as in the dermo-epidermal junction, presenting morphologically as layers of decreased thickness and fibroblast cells; reduced levels of GAGs (increased water skin loss); and an increased number of metalloproteinases [10,13,30]. It was shown that in the dermis of aged skin, there are more neutrophils and mast cells than in young individuals, which is correlated with upregulation of IL-8 [30]. Some studies showed that in the aged epidermis, there is a lower number of Langerhans cells and they are less willing to relocate to trauma/infection localization. A smaller count of dendritic cells is probably linked to a poorer response to vaccination in old age [10,14]. A higher ratio of CD4^+^ to CD8^+^ T cells found in aged skin also predisposes to inflammation [14].

Moreover, there is also an aging of the immune system that results in various processes such as the accumulation of senescent cells which, with time, leads to cell cycle blockade (moderated by p16 protein) [7,10,12,14,22,30]. An evaluation of aging skin showed that it has more senescent fibroblasts than young skin, and that its accumulation leads to an imbalance of skin homeostasis and causes poorer wound healing and the start of oncogenesis [10,29]. It has been shown that with aging, dermal fibroblasts release a lower level of IGF-1 which leads to poorer collagen synthesis and skin atrophy due to the increased accumulation of the DNA damage-induced phosphorylated histone protein, γH2AX, and p16INK4a-positive epidermal cells. In the case of photoaging, a higher amount of pro-melanogenic growth factors, including hepatocyte growth factor and SCF are encountered [11]. Senescent keratinocytes accumulate in the elderly [10], producing more IL-1α in comparison to young people. Aged melanocytes also showed elevated markers of inflammaging characteristic of skin atrophy due to the influence on basal keratinocytes (activation of CXC chemokine receptor 3-dependent mitochondrial ROS) [11]. Barth et al. reported that skin samples showed a greater modulation of cell cycle and senescence-associated genes than in other organs. They also found that age-related expressions of inflammaging have a lower impact on the skin than on the blood, liver, or brain samples [7]. However, they did not observe any characteristic genes responsible for skin aging.

Skin aging results in reduced skin flexibility and density (wrinkling), a reduced ability to heal or resist external pathogens or toxins, and an increased tendency to pigmentation disorders [7,9,10,11,13,19,23,30], dry skin [19], and telangiectasia [9]. There are studies on transepidermal water loss (TEWL). Some authors found a relationship between aging and higher TEWL rates (in some just on the decolette region). Furthermore, the TEWL rate was also higher among aged women than men. That process can cause epidermal disruption causing inflammaging [16]. Jarrod et al. analyzed photo-exposed skin samples from women (aged from 20 to 70 years) and described histological and morphological changes of aged skin such as epidermal thinning, rete ridge pathlength loss, and stratum corneum thickening. They hypothesize that a better outcome can be expected in slowing down inflammaging when anti-aging procedures are started at a younger age [29].

## 8. Summary

Skin aging is a complex process that is caused by many intrinsic and extrinsic factors that have not been fully elucidated. Aging processes can be divided into three levels—molecular, cellular, and tissue with some including inflammaging [18]. In this review, we discussed the definitions of inflammaging and immunosenescence which both contribute to skin aging. Several factors were mentioned in the literature to accelerate inflammaging. Some of them are modifiable, such as smoking, exposure to UV radiation, diet, or pollution, while others, such as the functioning of the immune system, are difficult to influence and understand. While knowledge on external factors can help people to improve their health conditions, knowledge on biochemical factors can help researchers to understand inflammaging process and develop interventions to minimize the impact of aging on the human body.

This review shows several gaps in the evidence that require further research. Knowledge on factors slowing down or accelerating inflammaging is insufficient. One of the potential areas of interest is the skin microbiome which, together with the gut microbiome, can contribute to the development of many acute and chronic lifestyle diseases.

This targeted review has some limitations owing to its limited scope and qualitative nature. We restricted our qualitative analysis to the most interesting articles and provide in-depth insight into the process of inflammaging. Furthermore, performing a targeted literature search only enabled us to define gaps while providing a broader insight into the topic. We also acknowledge that this method is not as comprehensive as a systematic review.

## 9. Conclusions

The entire process that causes inflammaging has not been fully elucidated. The evidence summarized in this review highlights many gaps in knowledge; however, studies that have been conducted to date have provided important data on inflammaging. Although the body of evidence on substances that can stop or slow down inflammaging is growing, researchers focus mainly on their systemic action and capabilities to provide longevity. Further research is recommended to continue studies on inflammaging with a greater focus on its impact and possible delay of aging within the skin.

## Data Availability

Not applicable.
